# Assessing the Effectiveness of Automated Emotion Recognition in Adults and Children for Clinical Investigation

**DOI:** 10.3389/fnhum.2020.00070

**Published:** 2020-04-07

**Authors:** Maria Flynn, Dimitris Effraimidis, Anastassia Angelopoulou, Epaminondas Kapetanios, David Williams, Jude Hemanth, Tony Towell

**Affiliations:** ^1^School of Social Sciences, University of Westminster, London, United Kingdom; ^2^School of Computer Science and Engineering, University of Westminster, London, United Kingdom; ^3^ECE Department, Karunya Institute of Technology and Sciences, Coimbatore, India

**Keywords:** emotion, brain, artificial neural network, computing, clinical investigation

## Abstract

Recent success stories in automated object or face recognition, partly fuelled by deep learning artificial neural network (ANN) architectures, have led to the advancement of biometric research platforms and, to some extent, the resurrection of Artificial Intelligence (AI). In line with this general trend, inter-disciplinary approaches have been taken to automate the recognition of emotions in adults or children for the benefit of various applications, such as identification of children's emotions prior to a clinical investigation. Within this context, it turns out that automating emotion recognition is far from being straightforward, with several challenges arising for both science (e.g., methodology underpinned by psychology) and technology (e.g., the iMotions biometric research platform). In this paper, we present a methodology and experiment and some interesting findings, which raise the following research questions for the recognition of emotions and attention in humans: (a) the adequacy of well-established techniques such as the International Affective Picture System (IAPS), (b) the adequacy of state-of-the-art biometric research platforms, (c) the extent to which emotional responses may be different in children and adults. Our findings and first attempts to answer some of these research questions are based on a mixed sample of adults and children who took part in the experiment, resulting in a statistical analysis of numerous variables. These are related to both automatically and interactively captured responses of participants to a sample of IAPS pictures.

## 1. Introduction

Emotions are the essence of what makes us human. Emotional response can be measured by at least three different systems: affective reports, physiological reactivity, and overt behavioral acts (Lang, 1969). One of the strongest indicators for our emotions has always been considered our face. Cross-cultural studies suggest that there is a set of universal basic emotions that can be recognized from facial expressions, including anger, disgust, fear, sadness, and enjoyment (Ekman, 1993). Facial expressions are a strong correlate of emotion, and it has been shown that almost everyone can produce and recognize facial expressions (Ekman and Friesen, 1978; Ekman, 2016). Consequently, previous studies have investigated emotional reactions using affective pictures to elicit emotional experience in adults (Greenwald et al., 1989) and in children (McManis et al., 2001).

Prominent position in these studies was taken by the International Affective Picture System (IAPS) (Lang et al., 1997), which provides a set of normative emotional stimuli for experimental investigations of emotion and attention. When used in combination with tools for the collection of subjective affective ratings such as the Self-Assessment Manikin (Bradley and Lang, 1994) or the Affective Slider (Betella and Verschure, 2016), which are non-verbal assessment techniques that directly measure the pleasure and arousal associated with a wide variety of stimuli, emotional affect can be measured. Furthermore, skin conductance is also a sensitive autonomic measure of emotional arousal (Boucsein et al., 2012). The higher the arousal, the higher the skin conductance for both positive (“happy” or “joyful”) and negative (“threatening” or “saddening”) stimuli. Consequently, biometric research platforms have emerged specializing in computer vision and machine learning techniques, which enable reliable, valid measurement of emotion-related facial expressions from real-time non-invasive sensors (Sikka et al., 2015). Combining computer machine learning techniques that measure facial expressions with skin conductance responses and self-report may provide useful insight into emotional states.

Despite all these technological advancements, there is currently an ongoing lively debate about the effectiveness of automated emotion recognition approaches. For instance, there seems to be a paradigm shift from the basic emotion perspective to an appraisal perspective to find the appropriate theory integration in the area of automated facial emotion classification. In general, the criticism of the basic emotion perspective argues that, although automated facial expression analysis may classify basic emotional expression categories, it might not ultimately measure emotional states.

The fact that automated facial expression analysis relies on the assumption that there is coherence between emotion and facial expressions (Bonanno and Keltner, 2004; Reisenzein et al., 2013) limits the interpretation of data generated by automated facial expression analysis and throws into question the generalization of automated emotion classification (Wolf, 2015). Furthermore, some researchers argue that inferences based on data generated by automated facial expression analysis should build upon emotion theories that go beyond the basic emotion perspective, adopt an appraisal perspective, and allow more flexibility to consider different contexts.

Further to this criticism, relying on machine learning techniques and algorithms also raises the question of whether the algorithmic design and implementation introduced is transparent and also discrimination- and fairness-aware. It is only then that classifications or predictions, such as those imposed by the foreseen recognition of emotions in children prior to clinical investigation, are trustworthy and not subject to bias. Generally speaking, there are two sources of bias to be prevented: (a) *data sources and input*, (b) *algorithms* (Hajian and Bonchi, 2016).

Given this context, this paper contributes to the lively debate and criticism surrounding the effectiveness of automated emotion recognition approaches. In particular, it presents the results and interesting findings from a study and experiment that set out to determine how children and adults may respond to emotional stimuli and whether such emotions can be adequately captured and analyzed by state-of-the-art biometric research platforms. The study has the potential to advance our ability to identify children in a hospital environment who are very anxious, scared, or upset.

This paper, however, focuses on the observed discrepancies, under specific circumstances, in expected (e.g., IAPS) and observed (e.g., biometric research platform, subjective classification) emotional responses, which may further help in identifying the root for the emergence of such a criticism against automated emotion recognition approaches, in general, and those based on facial recognition, in particular. Hence, the rest of the paper is structured as follows. The state of the art is reviewed in section 2. Section 3 presents our methodology and experimental setup, where materials are thoroughly explained by giving an overview and then focusing on each piece of the system. Then, the first statistical results are presented in sections 4.1 (adults) and 4.2 (children). Finally, we conclude by also considering the outlook for the future.

## 2. Related Work

This section provides a brief overview of the literature on the attempts to classify emotions and of the development of affective computer systems in relation to facial expression recognition and other computer-based systems developed to recognize human emotions.

Emotion refers to a shaking of the organism as a response to a particular stimulus (person, situation, or event), which is generalized and occupies the person as a whole. Usually, it is very brief in duration, which makes it different than Mood. Mood is a feeling that tends to be less intense than emotion and often lacks a contextual stimulus (Weiss and Cropanzano, 1996; Feidakis et al., 2011). Both emotions and moods are encompassed under the umbrella of “Affect,” which is a generic term that covers a broad range of feelings that people experience (George, 1996). Affective computing is the set of techniques aimed at performing affect recognition from data in different modalities and by using multiple sensors in order to increase the reliability of estimates. Affective computing involves two areas: emotion synthesis, which is used to artificially imitate some of the physical or behavioral expressions associated with affective states, and emotion analysis, which is often employed in decision making for interactive systems (Shen et al., 2009; Poria et al., 2017). In this paper, we discuss and implement both kinds of affects by specifically using well-established datasets such as the IAPS for emotion evocation and stimulus and facial recognition software for identifying and analyzing emotions in an adult and child population.

Before discussing the methodology of our affective system, we introduce the literature on the different categorizations of emotions and the state-of-the-art of affective systems in relation to protective groups. In the last several decades, psychologists have categorized emotions with two fundamental viewpoints: (1) discrete categories, and (2) emotions grouped on a dimensional basis. In the first category, all humans are believed to have an essential set of basic emotions that are distinguishable by an individual's facial expression and biological processes (Colombetti, 1995). Most emotion experts think there are a few “basic emotions,” although they do not all agree on what they are or why they are basic (Ortony et al., 1987). Ekman (2003) and Ekman and Friesen (1978) has been very influential with his studies of facial expressions. He developed a list of basic emotions that are universal, not culturally determined. These basic emotions are “anger, disgust, fear, happiness, sadness, and surprise” (Ekman and Friesen, 1978). A few tentative efforts to detect non-basic affective states, such as “fatigue, anxiety, satisfaction, confusion, or frustration” have also been made (Dalgleish and Power, 1999; Prinz, 2004). In the second category, researchers define emotions according to one or more dimensions. Most of these models integrate valence and arousal or intensity dimensions, as they propose that a common and interconnected neurophysiological system is responsible for all affective states (Rubin and Talerico, 2009). Russell 1980; 2003 circumplex model of affect was developed on the basis that affective states arise from cognitive interpretations of core neural sensations that are the product of two independent neuro-physiological systems. The model suggests that emotions are distributed in a two-dimensional circular space, valence and activation (or arousal). Valence represents the horizontal axis. It can be pleasant (positive) such as happiness or joy, or it can be unpleasant (negative), such as anxiety, anger, or boredom. Researchers have criticized two-dimensional models as being too simplistic. Recent evidence suggests there should be a fourth dimension (Fontaine et al., 2007). Watson and Tellegen (1985) changed the orientation and proposed four dimensions: “pleasantness, engagement, positive, and negative affect.” Fontaine et al. (2007) reported consistent results from various cultures where a set of four dimensions is found in user studies, namely “valence, potency, arousal, [and] unpredictability.” Plutchik (1980, 2003) proposed a cone-shaped model with intensity of emotional experience represented by depth, similarity by nearness, and four pairs of opposites, all represented by color-coded segments.

As we have seen from the above categorization of emotions, Ekman (2003); Ekman and Friesen (1978), a pioneer in the visual modality analysis of emotions, referred to facial expressions as primary cues for understanding emotions and sentiments. Facial expressions are a gateway into the human mind, emotion, and identity and, along with textual data, can provide important cues to better identify true affective states in the participants (Taggart et al., 2016; Kim et al., 2018). It can be crucial to understand facial characteristics when working with patients, especially patients who are unable to communicate in other ways, for example, when trying to assess emotions in children unable to self-report information. This is particularly true when the children have multi-systemic problems and may be dysmorphic, making interpretation of facial expressions even more difficult.

Therefore, in clinical environments, assessments of the child's emotional state are typically made by clinical staff or family members. However, in some instances, staff may have difficulty in accurately estimating children's emotional states, and family members/carers may not always be available. In such cases, automated systems based on computer vision and machine learning techniques that can reliably process and analyze valid measurements of emotion-related facial expressions without using invasive sensors can play a crucial role in diagnostic cases such as autism. Due to the nature of these studies, which have very detailed ethical requirements and require access to data on protected groups, only a handful of studies have examined the efficacy of automated systems in detecting emotional expressions in individuals from protected groups in order to assist and define protocols for better therapeutic treatments. Trevisan et al. (2016) used facial expression analysis technology to determine how children with and without autism spectrum disorder (ASD) may differentially produce facial expressions in response to emotional stimuli and whether alexithymia may contribute to diminished facial expressions. Xefteris et al. (2016) developed a methodology for emotion recognition using facial expressions as indicators to evaluate the overall health status of subjects suffering from neurodegenerative diseases (e.g., Mild Cognitive Impairments, Alzheimer's, dementia). Leo et al. (2015) used machine learning strategies based on facial expressions during robot-child user interaction to evaluate the behaviors of children who belong to the ASD group for the development of better therapeutic protocols. Suzan and Mariofanna (2016) used computer vision and machine learning methods such as active shape models (ASM) and Support Vector Machine (SVM) to recognize facial expressions in children with ASD during playtime. Kunz et al. (2017) used an interdisciplinary approach of human observers and video-based pain detection systems that analyzes facial expressions to identify pain in people with dementia and ensure effective treatment and ongoing care.

In addition, studies using physiological signals to recognize emotional states such as electroencephalogram (EEG)-based brain-computer interface systems (BCI) are also providing interesting results, and there is promise for use in a number of real-world applications. Huang et al. (2019) showed participants video clips with negative and positive valence while recording EEG. The EEG-based BCI system successfully induced and recognized positive and negative emotional states in patients with Disorders of Consciousness. Hou and Chen (2019) presented a system for characterizing emotions using EEG signals, where four classes of emotions in particular (i.e., happy, sad, calm, and angry) could be distinguished. They induced these emotions by musical stimuli (using 20 music passages in each music emotion group) and recorded the EEG signals of the subjects using 12 electrodes. Guan et al. (2019) proposed a novel classification framework using a decision tree (DT) classifier to distinguish between multiclass motor imagery (MI) for BCI. Their proposed data reduction method performed better when compared to state-of-the-art semisupervised joint mutual information (semi-JMI) and general discriminant analysis (GDA) methods. Fernàndez-Rodríguez et al. (2019) used different sets of flashing stimuli in a number of participants in order to assess the effect of the emotional stimuli in these images by using a P300 brain-computer interface (BCI) speller. Finally, it has been demonstrated in various studies (Acharya et al., 2018; Jahmunah et al., 2019) that EEG signals are commonly used to detect brain diseases such as depression and schizophrenia.

All of the above models of emotions are very important when designing and informing the development of affective systems. Dimensional models have been used by various researchers, mainly because they provide a way of describing a wide range of emotional states that occur in real life. “Basic” emotional models were very influential in early human-computer interaction studies. When all these emotional models are put into a computational framework where programmers and developers map aspects of emotion to aspects of the system, different models have different pros and cons (Bosse et al., 2010). In our study, we have used basic emotional models in the categorical data and dimensional models for automatically measuring and analyzing the emotions of related behaviors.

## 3. Methodology and Experimental Design

In the following sections, two experiments will be described, one with adult participants and one with child participants. Studies such as Mikels et al. (2005), which aimed to provide categorical data for the IAPS dataset, have shown that some pictures were rated as evoking a combination of emotions, for example, anger, fear, and disgust. Studies such as Barke et al. (2012) have shown that there are sex differences and cross-cultural differences in the rating and categorization of a subset of IAPS pictures, with women tending to rate negative pictures more negatively and with higher arousal ratings than men, and they established valence and arousal norms for a German sample, suggesting that country and sex-specific norms should be used when selecting IAPS pictures.

Due to the paucity of studies providing categorization of IAPS pictures in a British sample, in the current adult study, pictures were selected from the Mikels et al. (2005) paper that had been rated as representing sadness and fear only, not mixed emotions, and only those that in their sample did not show gender differences in their valence and arousal ratings. Due to the small sample size in both our studies, sex differences and cross-cultural differences in ratings were not taken into account.

The nature of work for this research is rooted in empirical software engineering using a controlled experiment method. The system, which will recognize the participants' emotions and control the materials delivery, is the independent variable that will be manipulated to measure its effect on the dependent variable, which will be the participants' emotional state during the assessment.

### 3.1. Participants

For the adult-related experiment, nineteen participants were recruited, the demographics of whom are depicted in Table 1. Participants were a combination of undergraduate psychology students from the University of Westminster, who were awarded 1 h of research participation credit, and colleagues and acquaintances recruited by word of mouth. For the child-related experiment, eleven children were recruited (five females, six males, with a mean age of 11.5 years, SD 3.24, and an age range of 7–16). They were recruited by opportunistic sampling and word of mouth. Written parental consent and verbal child consent was obtained.

**Table 1:** Mean age and standard deviation for 19 participants.

	**Males (*n* = 8)**	**Females (*n* = 11)**
Age, years, mean ± SD	33.10 ± 16.06	28.55 ± 10.48
Age range in years	19–61	19–46

### 3.2. Ethics and Regulatory Framework

This study was carried out in accordance with the Ethics Code of the University of Westminster. This includes the assurance that data about an individual will be held securely, handled in accordance with the Data Protection Act 1998, and disposed of in line with Westminster's retention policy. The Ethics Code^1^ and the Data Protection Policy^2^ are available from the University of Westminster's Website. For the child-related experiment, written parental consent and verbal child consent was obtained.

In order to protect research participants and research staff involved with unpleasant IAPS protocols, specific measures were taken to ensure that the risk of negative psychological consequences was minimized for all parties involved, and the nature of the images shown was fully explained to both research staff and participants. As the researchers were interested in emotions that may be evoked in a hospital waiting room environment, no images involving mutilations or sexually arousing images were included, and the unpleasant images selected for the study had been identified as more likely to have been rated as evoking discrete emotions such as fear and sadness.

The researchers observed each participant during the study, and there were procedures in place so that if it was noticed that a participant was becoming distressed or emotionally upset, the researchers would ask the participant if they needed anything or would like to take a break. If a participant was distressed, the researcher would also get in contact with them later in the day to confirm that they were feeling less distress and that they had sought any help they may have needed. In addition, participants were provided with details of the University Counseling Services and, following completion of the study, all participants were fully debriefed.

#### 3.2.1. Data

Participants are referred to using unique numbers, and no collected data contains participant-identifying information. The consent form requires a signature and/or the initials of the participant, but this is kept separately from any data and is held in a secure file that is only available to the research team. Data are stored on a password-protected computer on University premises, and only the research team have access. Data stored on the laptop and external hard-drive are encrypted. Only members of the research team have access to the key. Participants had been made aware that their facial expressions will be photographed and videoed. No photographs or videos will be published without the participant's explicit consent.

### 3.3. Materials

#### 3.3.1. Photographic Stimuli

The International Affective Picture System (IAPS) (Lang et al., 1997) is a set of more than 900 standardized pictures that has been widely used in the study of emotion and attention, with more than 2700 citations. When used with tools for the collection of subjective affective ratings such as the Self-Assessment Manikin (SAM) (Lang et al., 1997) or the Affective Slider (Betella and Verschure, 2016), insights into the dimensional aspects of emotion are derived. The set of pictures includes pictures such as snakes, accidents, kittens, babies, and everyday items such as chairs. Based on previous emotional ratings, 80 pictures were selected for the current study, 50% neutral, 10% pleasant, and 40% unpleasant. The pictures selected were based on a study by Mikels et al. (2005) who attempted to provide categories for the IAPS pictures based on the ratings of pictures by a sample of 120 participants in the United States. Although it can be difficult to elicit emotional responses in a laboratory environment, particularly discrete emotions, the pictures in the current study were selected to try to evoke emotions in a hospital waiting room environment, so pictures that were rated as likely to represent sadness and fear were chosen as well as those rated for happiness. Pictures in the dataset that had been rated as showing a mixture of emotions, e.g., fear, anger, and anxiety were excluded as were pictures that had shown sex differences in their ratings.

Normative ratings are available for the pictures based on a nine-point scale. Mikels et al. (2005) selected the images based on minimum criteria. The negative images met the minimum criterion that they be less than the neutral midpoint of 5 (mean pleasure rating = 3.05, *SD* = 0.84, and mean arousal rating = 5.56, *SD* = 0.92). The positive subset were selected as positively valenced on the criterion of being equal to or greater than 5 (mean pleasure rating = 7.05, *SD* = 0.63, mean arousal rating = 4.87, *SD* = 0.98). As we were particularly interested in negative emotions, the images selected were based on emotions that may be experienced in a hospital waiting room, such as fear and sadness. Images of a sexual nature or ones that evoked disgust were not relevant to the current study.

#### 3.3.2. Affective Digital Slider

**Figure 1:**
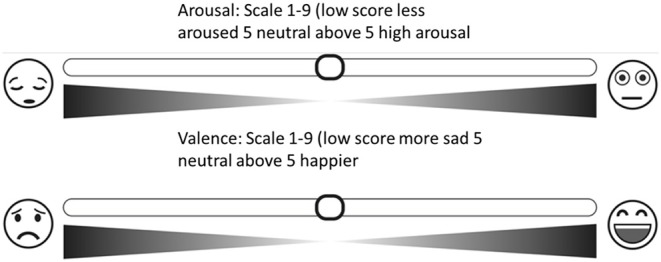
Overview of the study protocol including the “Affective Slider” (Betella and Verschure, 2016) (AS), which measures arousal (top) and pleasure (bottom) on a continuous scale.

The affective digital slider is a tool that provides a self-assessment scale for the measurement of human emotions that does not require written instructions. There are two sliders, one measuring arousal, ranging from calm to excited, and one measuring affective valence, ranging from pleasant to unpleasant (see Figure 1). Each slider measures a single value on a continuous normalized scale ranging from 1 to 9 with a central value equal to 5 and a minimum resolution 0.01; 9 represents a high rating on each dimension (i.e., high pleasure, high arousal) and 1 represents a low rating on each dimension (i.e., low pleasure, low arousal).

#### 3.3.3. Subjective Data

In addition to providing dimensional data using the digital slider, participants were asked to select from a list the word that best described the predominant emotion that they felt after viewing each picture. The list included the words happy, sad, fear, neutral, and disgust; there was also an option for the participants to select ‘other' and write their own description. For data analysis purposes, these subjective ratings were then coded as negative, neutral, or positive (such that happy was rated as positive, sad, fear were negative).

### 3.4. Biometric Research Platform

**Figure 2:**
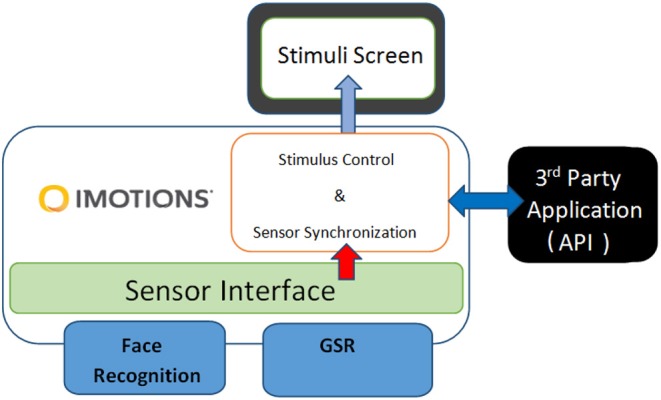
System diagram of the multimodal human behavior study.

Galvanic Skin Response (GSR) and facial expressions were measured using the iMotions Biometric Research Platform 6.0, iMotions A/S, Copenhagen, Denmark, 2016. An Application Programming Interface (API) module was used together with the iMotions platform and the Galvanic sensor to monitor and control in real time the connections with the biometric sensors through TCP ports, and the data flow of the experiment, with time, sequence number, and stimulus name and type assigned to variables (see Figure 2). Facial expressions were recorded for analysis via a webcam (Logitech HD Pro Webcam C920).

**Figure 3:**
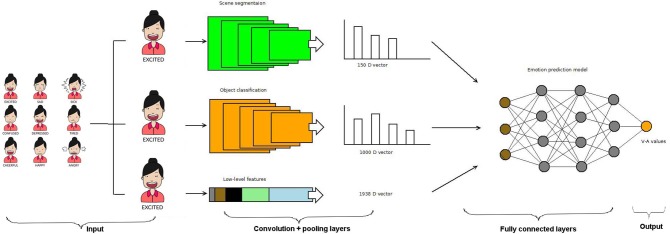
The general pipeline of the iMotions recognition system. Adapted from Kim et al. (2018). Female Mood Avatars by Namnso Ukpanah^3^.

iMotions can detect changes in key face features such as brows, eyes, and lips and analyze the basic emotions of the recorded face. Researchers can choose between two different algorithms to classify emotions from facial expressions in iMotions's platform: the FACET module, based on the FACET algorithm (Littlewort et al., 2011), and the AFFDEX module, based on the AFFDEX algorithm by Affectiva Inc. (El Kaliouby and Robinson, 2005; McDuff et al., 2010). Affectiva is an API for emotion recognition using deep learning. It is said to have nearly 6 million faces as an emotion database in order to provide great accuracy^4^. These algorithms detect facial landmarks and apply a set of rules based on psychological theories and statistical procedures to classify emotions (Li and Deng, 2018; Stöckli et al., 2018). Different algorithms, like AFFDEX and FACET, use distinct statistical procedures, facial databases, and facial landmarks to train the machine learning procedures and ultimately classify emotions (Kim et al., 2018). For all our experiments, we have used the AFFDEX algorithm. iMotions classifies the seven basic emotions (joy, anger, surprise, fear, contempt, sadness, and disgust) and provides a confidence rating for the probability that an emotion is being expressed. For data analysis purposes, joy was coded as positive; anger, fear, contempt, sadness, and disgust were coded as negative; and data reflecting surprise was excluded, as surprise can be either positive or negative in valence. Figure 3 shows the iMotions architecture diagram with an image or video sequence as input, the feature maps based on convolution and pooling layers, the fully connected layers, and the output, which can be happiness, sadness, or any of the seven emotions classified by iMotions.

Galvanic skin response was measured from the phalanx of the index and middle finger of the nondominant hand using 1 *cm*^2^ Ag/AgCl (silver/silverchloride) electrodes placed in reusable snap-on Velcro straps. For each participant, the GSR recorded in microvolts (μ*V*) was segmented into 8-s intervals for each picture, and the mean of each participant's 2-min baseline measure was subtracted from the peak of each segment.

**Figure 4:**
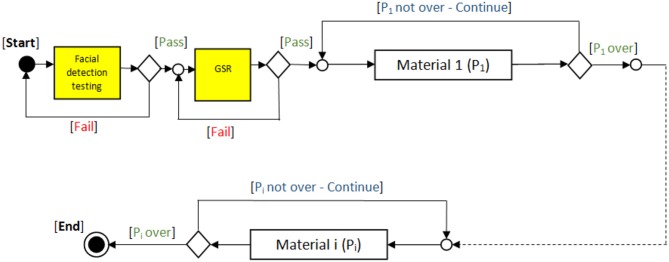
UML interaction overview diagram.

The API module was designed to receive the biometric sensor data, analyse it, and control the delivery of the presented materials. The Unified Modeling Language (UML) interaction overview diagram (see Figure 4) shows how the system starts by testing the biosensors, starting with the camera for facial detection, then the GSR sensor. After passing the tests successfully, the participant engages with the first material (P1), while the API reads and analyses the data provided by the sensors. The material will continue playing to the end before moving to the next. This process will continue in the same pattern through the rest of the materials.

**Figure 5:**
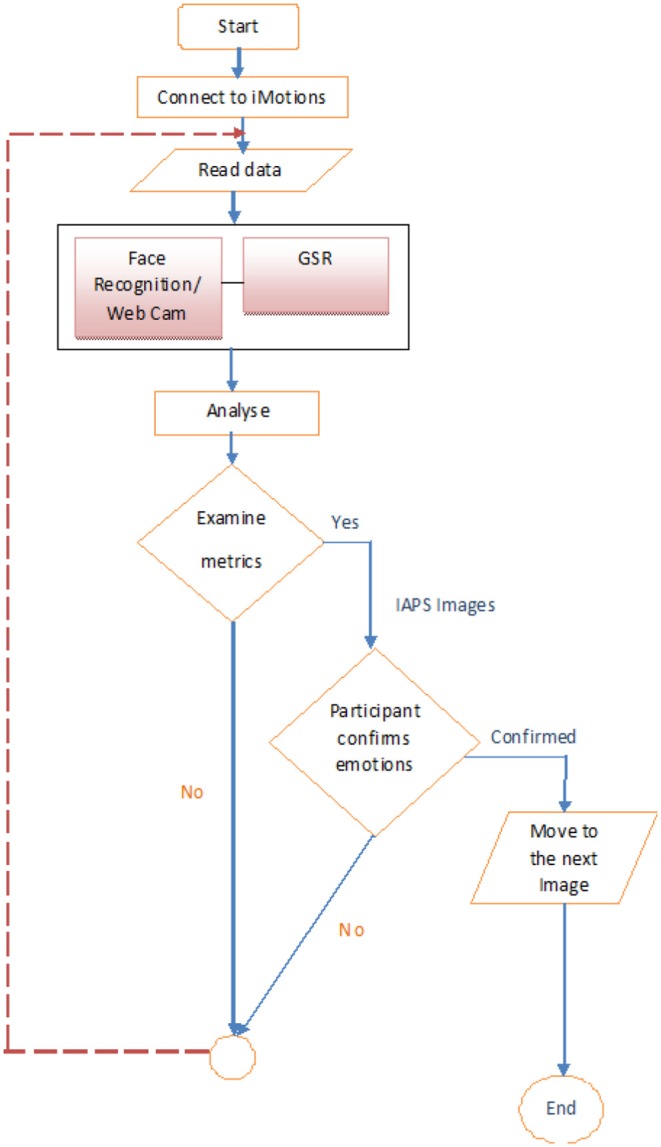
Flowchart of the pilot study of automated emotion assessment in adults and children.

Figure 5 presents a flow chart for the pilot study of the automated emotion assessment in adults and children. The API continuously reads/monitors the data and provides control signals accordingly until the emotion assessment session is completed.

### 3.5. Protocol

Participants were seated comfortably in front of a computer screen. They were advised that their facial expressions in response to each photograph would be recorded via a webcam and recorded with iMotions facial expression analysis software. Participants were recorded individually in a quiet laboratory at the university and were asked to rate a set of 80 photographs selected from IAPS. The order of the photographs was pseudo-random such that each emotive photograph was preceded by a neutral photograph. Each photograph was presented on a computer screen for 8 s. Participants were instructed to maintain their attention on the screen for the whole time that the image was present. Galvanic skin response was recorded from each participant at baseline for 2 min, during which time the participants were asked to relax and close their eyes. GSR was then recorded throughout the study. Standardized instructions were read to each participant based on the Self-Assessment Manikin instructions (Bradley and Lang, 1994) amended for use with the Affective Slider (Betella and Verschure, 2016) with the instruction to “move the sliders to express how you actually felt while watching the picture.” Participants were asked to view and rate four photographs that were similar to those used in the study to familiarize themselves with the rating scales and to ensure that they were happy to participate. Written consent forms were completed.

**Figure 6:**
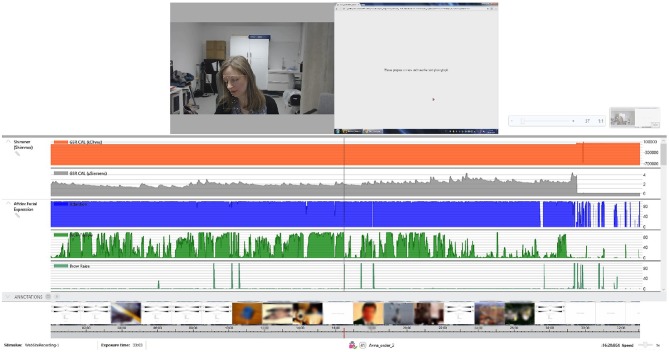
Overview of the study protocol. The top row shows the participant together with the facial landmark points assigned by the iMotions facial recognition algorithm and a screen with instructions. The middle row shows the GSR and the Affdex Facial expression metrics. The bottom row shows in a timeline the response of the participant per image, and the Affective Slider, which, as can be seen in Figure 1, measures arousal (top) and pleasure (bottom) on a continuous scale. Due to copyright issues associated with the IAPS images, all images at the bottom have been blurred.

Figure 6 shows the experimental set up with the participant. Following each photograph, the screen showed the rating page, and the participant was asked to provide their subjective affective ratings for pleasure and arousal using the Affective Slider and then to select the word that indicated the predominant emotion that they felt when viewing the picture. Participants were advised that if the word that described their emotion was not there, they should select ‘other' and type the word that best describes how they felt in response to the photograph in the space provided. The rating screen stayed in place until ratings had been made. A further screen then appeared for 4 s, advising the participant to prepare to view the next slide.

#### 3.5.1. Additional Notes for the Child-Related Experiment

Each child was rewarded for their participation with a £10 “Love to Shop” voucher. They were advised that they could withdraw their participation at any point without penalty. Recording of the data took place in the participants' home environment. Thirty-two images (16 neutral, eight positive and eight negative) were selected from IAPS for the study with children based on those used in a study by McManis et al. (2001), where they had been judged by teachers to be appropriate for viewing by children in the age range 7–14 years. The images covered a wide range of affective content, and each emotive image was preceded by a neutral image. Standardized instructions based on the SAM for children, adjusted for use with the Affective Digital Slider, were read to each participant. Participants were seated in front of a laptop with a built-in camera that recorded their facial expressions. Parents were allowed to be in the room if they requested to be.

## 4. Statistical Analysis and Results

The following section provides details of the treatment of results from both experiments. The participant's valence, arousal, and GSR scores are subjected to Analysis of Variance (ANOVA), whilst participants' subjective ratings and iMotions classification are subjected to analysis using Chi-square.

### 4.1. Study With Adults

Participants' responses on the digital slider scales for valence and arousal were recorded for each picture, as was GSR response. Mean scores and standard deviations (SD) of participants' ratings (on a scale of 1-9) for each of the IAPS negative, neutral, and positive pictures and their GSR response recorded in microvolts (μ*V*) are shown in Table 2. GSR data for one participant was excluded due to technical difficulties during the recording.

**Table 2:** Mean (+SD) valence and arousal ratings for the Affective Digital Slider (*n* = 19) and GSR data (*n* = 18) for each IAPS picture category.

	**IAPs category**		
**Measure**	**Negative (*n* = 30)**	**Neutral (*n* = 40)**	**Positive (*n* = 10)**
Valence	3.49 (±0.91)	5.02 (±0.23)	6.38(±1.19)
Arousal	5.78 (±1.13)	4.55 (±1.03)	4.16 (±1.00)
GSR(μV)	9.16 (±27.67)	8.78 (±27.50)	11.73 (±28.02)

Low scores (i.e., < 5) indicate negative valence and low arousal, scores around 5 indicate neutral valence and little or no reported arousal, and higher scores (i.e., >5) indicate positive valence and high arousal. The descriptive statistics reported in Table 2 suggest that, for valence, as expected, negative pictures have the lowest mean score, suggesting that they evoked negative emotions, neutral pictures have a mean score at the mid-point of the scale, suggesting that neither positive or negative emotion was evoked, and positive pictures have the highest mean score, suggesting that more positive emotions were reported.

In terms of arousal, it was expected that both positive and negative images would have a higher mean arousal rating than neutral images. However, the descriptive statistics suggest that negative images elicited a higher mean arousal rating, while the ratings for neutral and positive images were similar. In addition, mean GSR scores (μ*V*) indicate that positive pictures and negative images elicited a greater GSR response than neutral pictures, with positive pictures eliciting the greatest GSR.

In order to establish whether any of these differences were significant, repeated-measures one-way ANOVA was conducted. The within-subjects factor, the type of IAPS image, had three levels: negative, neutral, and positive. There was a significant effect of the type of IAPS image on the valence score [*F*_(2, 36)_ = 39.989, *p* < 0.01]. Bonferroni-corrected simple effects reveal that, using the digital slider, positive images were rated as significantly more positive than both neutral and negative images (*t* = 4.871, *df* = 18, *p* < 0.05; *t* = 6.538, *df* = 18, *p* < 0.05) and negative images were rated as significantly more negative than neutral images (*t* = 1.420, *df* = 18, *p* = 0.173).

There was a significant effect of the type of IAPS image on the arousal score [*F*_(2, 36)_ = 10.500, *p* < 0.01]. Bonferroni-corrected simple effects reveal that, using the digital slider, negative images were rated as significantly more arousing than positive images (*t* = 3.872, *df* = 18, *p* = 0.001) but not significantly more arousing than neutral images (*t* = 3.093, *df* = 18, *p* = 0.006), and there was not a significant difference in arousal rating between neutral and positive images (*t* = 7.636, *df* = 18, *p* < 0.05).

There was a statistically significant effect of the type of IAPS image on the GSR score [*F*_(2, 34)_ = 25.037, *p* < 0.001]. Bonferroni simple effects reveal that positive images provoked a significantly higher GSR than neutral or negative images (*t* = 5.387, *df* = 17, *p* = 0.001; *t* = 4.758, *df* = 17, *p* < 0.001); there was no significant difference in GSR between neutral and negative images (*t* = 2.341, *df* = 17, *p* = 0.032). These findings suggest that, when using the digital sliders, participants generally showed agreement with the IAPS classification of the images in terms of their valence rating and that negative images were more arousing when rated using the digital slider but that positive images elicited the greatest GSR.

As ratings using the affective digital slider show that participants generally rate the images in accordance with the IAPS classification and neutral images were only included in the study to bring participants' valence and arousal back to neutral between each of the emotive images, analysis of the association of subjective ratings and picture type and iMotions data and picture type focused on the negative and positive images.

A Chi-squared test was conducted to test for an association between the type of image displayed (negative or positive) and subjective rating of participants, who selected a word that best described how each picture made them feel (negative or positive). Results show a significant association between IAPS picture type and subjective rating (*X*^2^ = 192.700, *df* = 1, *p* < 0.001). Participants rated negative pictures as negative in 85.6% of cases and rated positive pictures as positive in 75.9% of cases (see Table 3 for observed and expected counts). The results show that participants were more likely to rate a negative picture as negative and a positive picture as positive.

**Table 3:** Participants' ratings (negative, positive) of negative and positive images.

		**IAPS picture type**		
**Subjective rating**	**Count**	**Negative**	**Positive**	**Row totals**
Negative	Observed	357 (85.6%)	35 (24.1%)	392
	Expected	290.9	101.1	
Positive	Observed	60 (14.4%)	110 (75.9%)	170
	Expected	126.1	43.9	
Columns totals		417	145	562 (grand total)

The results of a Chi-squared test for an association between the type of picture displayed and the classification of the facial expression by iMotions software show a significant relationship between IAPS picture type and iMotions (*X*^2^ = 32.233, *df* = 1, *p* < 0.001). The iMotions software identified negative facial expressions in participants viewing negative images in 95.8% of cases and identified positive facial expressions in participants viewing positive images in 23% of cases. Interestingly, iMotions also identified negative emotional reactions to 77% of the positive pictures (see Table 4 for expected and observed counts). The results show that iMotions software was more likely to identify negative facial expressions in response to negative images but interestingly, more negative facial expressions to positive pictures.

**Table 4:** iMotions classification (negative, positive) of participants' facial expressions to negative and positive images.

		**IAPS picture type**		
**iMotions identifier**	**Count**	**Negative**	**Positive**	**Row totals**
Negative	Observed	277 (95.8%)	77 (77%)	354
	Expected	263.0	91.0	
Positive	Observed	12 (4.2%)	23 (23%)	35
	Expected	26.0	9.0	
Columns totals		289	100	389 (grand total)

### 4.2. Study With Children

Participants' responses on the digital slider scales for valence and arousal were recorded for each picture, as was GSR response. Mean scores and standard deviations (SD) of participants' ratings (on a scale of 1-9) for each of the IAPS negative, neutral, and positive pictures and their GSR response recorded in microvolts (μ*V*) are shown in Table 5.

**Table 5:** Mean (+SD) valence and arousal ratings for the Affective Digital Slider (*n* = 11) and GSR data for each IAPS picture category.

	**IAPs category**		
**Measure**	**Negative (*n* = 8)**	**Neutral (*n* = 16)**	**Positive (*n* = 8)**
Valence	5.26 (±0.49)	5.01 (±0.42)	5.40 (±0.67)
Arousal	5.62 (±0.67)	4.43 (±1.13)	5.87 (±0.87)
GSR(μV)	3994.85 (±203.60)	3997.65 (±204.59)	3999.27 (±204.16)

The descriptive statistics reported in Table 5 suggest that, for valence, the pictures did not evoke reportable negative or positive emotions. In terms of arousal, both positive and negative images have a higher mean arousal rating than neutral images. In addition, mean GSR scores (μ*V*) indicate that positive pictures elicited a greater GSR response than both neutral and negative pictures. To establish whether any of these differences were significant, repeated-measures one-way ANOVA was conducted.

The within-subjects factor, the type of IAPS image, had three levels, negative, neutral and positive. There was no significant effect of the type of IAPS image on the valence score [*F*_(2, 20)_ = 1.744, *p* = 0.200]. This indicates that the pictures did not evoke reported emotional responses in the children. There was a significant effect of the type of IAPS image on the arousal score [*F*_(2, 20)_ = 6.028, *p* < 0.01]. Bonferroni-corrected simple effects (*p* should be ≤0.0016 to reach significance), however, did not reach significance when comparing negative with neutral images (*t* = 2.390, *df* = 10, *p* = 0.038) and positive with neutral images (*t* = 2.654, *df* = 10, *p* = 0.006).

There was a statistically significant effect of the type of IAPS image on the GSR score [*F*_(2, 20)_ = 22.193, *p* < 0.001]. Bonferroni-corrected simple effects reveal that positive images had a significantly higher GSR than negative images (*t* = 5.442, *df* = 10, *p* < 0.001), but there was no significant difference in GSR between neutral and negative images (*t* = 3.804, *df* = 10, *p* < 0.03) or between neutral and positive images; (*t* = 4.758, *df* = 17, *p* < 0.002). These findings suggest that, when using the digital sliders, participants did not report either negative or positive responses or higher arousal to negative and positive images, as would be expected. GSR, however, was higher in response to positive images.

In order to explore whether subjective ratings and iMotions measures of facial expression were associated with the type of picture presented, Chi-squared tests were conducted. A Chi-squared test was conducted to test for an association between the type of image displayed (negative or positive) and subjective rating of participants, who selected a word that best described how each picture made them feel (negative or positive). The results show no association between IAPS picture type and subjective ratings (*X*^2^ = 0.006, *df* = 1, *p* = 1). Participants rated negative pictures as negative in 50% of cases and rated positive pictures as positive in 49.4% of cases (see Table 6 for observed and expected counts). The results show that participants were just as likely to rate a negative picture as positive and a positive picture as negative.

**Table 6:** Participants' ratings (negative, positive) of negative and positive images.

		**IAPS picture type**		
**Subjective rating**	**Count**	**Negative**	**Positive**	**Row totals**
Negative	Observed	33 (50%)	40 (50.6%)	73
	Expected	33.2	39.8	
Positive	Observed	33 (50%)	39 (49.4%)	72
	Expected	126.1	43.9	
Columns totals		66	79	145 (grand total)

The results of a Chi-squared test for an association between the type of picture displayed and the classification of the facial expressions by iMotions software did not show an association between IAPS picture type and iMotions (*X*^2^ = 2.716, *df* = 1, *p* = 0.112). The iMotions software identified negative facial expressions in participants viewing negative images in 71.4% of cases where a negative picture was shown and identified positive facial expressions in participants viewing positive images in 46.7% of cases (see Table 7 for expected and observed counts). The results show that iMotions software was more likely to identify negative facial expressions in response to negative images but also to positive pictures.

**Table 7:** iMotions classification (negative, positive) of participants' facial expressions to negative and positive images.

		**IAPS picture type**		
**iMotions Identifier**	**Count**	**Negative**	**Positive**	**Row totals**
Negative	Observed	25 (71.4%)	24 (53.3%)	49
	Expected	21.4.0	27.6	
Positive	Observed	10 (28.6%)	21 (46.7%)	31
	Expected	13.6	17.4	
Columns totals		35	45	80 (grand total)

## 5. Discussion

The findings in the current study are interesting for a number of reasons. Rating the IAPS images using the Affective Digital Slider produced differing results for adults and children. Adults rated positive images with a higher valence, which was representative of the images making them feel happier than neutral images and negative images with lower valence, suggesting that the negative images made them feel sad/fearful. This is what would be expected and would appear to corroborate the IAPS classification of the images, as has been shown in many previous studies. However, it should be noted that the mean ratings for the positive images, although consistently higher than neutral, were not that much higher. In contrast, the children did not rate the negative, neutral, or positive images as differing in valence or arousal. These findings are in alignment with those of Vetella and Verscure (2016), who also found that standardized sets of stimuli, such as IAPS, may not be as effective as they once were at evoking emotions, due to our exposure to highly arousing stimuli in the media and general desensitization. In addition, the respective associations of subjective ratings and iMotions classification of facial expressions with picture category suggest that, for adults, subjective rating is better at identifying emotions than biometric software. It is unsurprising that participants show higher reliability than iMotions in classifying positive and negative images, given the importance of facial expression to human communication. In children, subjective ratings and the iMotions identification of expressions were at the level of chance. An additional factor that must be considered is that researchers in both studies noted little change in the facial expression of participants as they observed the IAPS images, once again suggesting that the stimuli may not have sufficiently evoked the emotions of the participants.

Other explanations for the difference in findings between children and adults could be due to the IAPS pictures selected and the environment in which the studies took place. Children were shown different and fewer images to ensure that the images were age-appropriate and that the task was not too onerous. The adult experiments took place in a laboratory at the University, whilst the children performed the task in their home environment. In the home environment, conditions such as lighting could not be controlled in the same way as in the laboratory, and this may have led to the biometric platform not detecting all facial movements. One of the difficulties with evoking and categorizing emotions in a laboratory scenario and not only through the use of pictures is establishing how each picture will actually make the individual feel—this will vary between participants. What may evoke fear in one participant may evoke anger, or indeed a mixture of more than one emotion, in another. In future studies, it is important to use more provocative stimuli (for example, emotive video clips) to ensure that emotions are sufficiently elicited and a larger sample and to ensure maximal environmental conditions for the use of the biometric platform.

While the biometrics facial recognition industry has grown, facial movements and expressions may not always be a reliable indication of how someone is feeling. Studies have shown that humans make assessments about other people's emotions based on factors including body language and tone of voice. As such, many emotion detection algorithms that have been developed in the last two decades are still facing problems with accuracy, complexity, and real-world implementation due to the irregularities in the complexity of models and unpredictability between expression categories. These approaches should thus always be used responsibly, especially when used in crime-detection applications.

## 6. Conclusion

In this paper, we contributed to the lively debate about and criticism of the effectiveness of automated emotion recognition approaches by attempting to question the following aspects: (a) the adequacy of well-established techniques such as the International Affective Picture System (IAPS), (b) the adequacy of state-of-the-art biometric research platforms, (c) the extent to which emotional responses may be different between children or adults. Our initial statistical analysis and results indicate that although there is, in general, an alignment between expected (IAPS) and observed (iMotions) responses for negative images, there is an interesting discrepancy in the expected and observed responses for positive images. This may be for many reasons ranging from incorrect classification of images in IAPS to incorrect classifications of responses by the biometric research system, iMotions, to significant changes in the emotional responses of the human population. In the future, we plan to dig deeper into the correlations among all of the significant variables by addressing all aspects of the experiment: facial recognition, subjective classification of responses, collection of images (IAPS), and different groups, i.e., adults and children. This multi-dimensional, multi-variate analysis will help shed more light on the real causes of such problems with automated emotion recognition, as well as into the limitations of current state-of-the-art approaches and technologies.

## Data Availability Statement

The datasets generated for this study are available on request to the corresponding author.

## Ethics Statement

This study was approved by the Psychology Ethics Committee at the University of Westminster. The participants provided their written informed consent to participate in this study. For those under the age of 16, written informed parental consent and verbal child consent was obtained. Written informed consent was obtained from the individual(s) AND/OR minor(s)' legal guardian/next of kin for the publication of any potentially identifiable images or data included in this article.

## Author Contributions

All authors contributed equally for the experimental analysis and preparation of the manuscript.

### Conflict of Interest

The authors declare that the research was conducted in the absence of any commercial or financial relationships that could be construed as a potential conflict of interest.

## Footnotes

^1^https://www.westminster.ac.uk/sites/default/public-files/general-documents/research-code-of-good-practice.pdf

^2^https://www.westminster.ac.uk/about-us/our-university/corporate-information/information-compliance-records-management-and-information-security/personal-data-protection/

^3^https://blog.affectiva.com/the-worlds-largest-emotion-database-5.3-million-faces-and-counting

^4^https://search.creativecommons.org/photos/21d1d4c8-a166-4ac2-9100-f6e33ab7b316
